# Association between *Faecalibacterium prausnitzii* Reduction and Inflammatory Bowel Disease: A Meta-Analysis and Systematic Review of the Literature

**DOI:** 10.1155/2014/872725

**Published:** 2014-03-27

**Authors:** Yuan Cao, Jun Shen, Zhi Hua Ran

**Affiliations:** Renji Hospital, School of Medicine, Shanghai Jiao Tong University, Shanghai Institution of Digestive Disease, Shanghai Inflammatory Bowel Disease Research Center, Shanghai 200127, China

## Abstract

*Background*. Laboratory data suggests a reduction of *Faecalibacterium prausnitzii* (*F. prausnitzii*) is confirmed both in fecal samples in inflammatory bowel disease (IBD) patients. Numerous observational studies have suspected dysbiosis, an imbalance between protective and harmful bacteria to be relevant to the etiology and pathogenesis of IBD. *Methods*. Medline, EMBASE, Pubmed, and others. were searched by 2 independent reviewers. Of 48 abstracts reviewed, 11 studies met our inclusion criteria (subject *N* = 1180). Meta-analysis was performed with Review Manager 5.2. *Results*. The bacterial count of *F. prausnitzii * in IBD patients was significantly lower (6.7888 ± 1.8875) log10 CFU/g feces than healthy controls (7.5791 ± 1.5812) log10 CFU/g feces; P < 0.0001. The Standardization Mean Difference of *F. prausnitzii* in IBD patients was −0.94 (95% confidence interval [CI]: −1.07–−0.80). Subgroup analyses revealed a trend toward a greater effect for CD (SMD: −1.13, 95% CI: −1.32–−0.94) when compared to UC (SMD: −0.78, 95% CI: −0.97–−0.60). *Conclusions*. The abundance of *F. prausnitzii* was decreased in IBD patients compared with healthy controls. Furthermore, the reduction of *F. prausnitzii* and misbalance of the intestinal microbiota are particularly higher in CD patients with ileal involvement.

## 1. Introduction 

IBD is suspected to arise from the interaction between the host's genetic background, mucosal immunity, and the resident bacterial flora [1]. Genome-wide association studies (GWAS) have identified more than 160 host genetic variants. Many are related to human gut microbiota [2]. In patients with inflammatory bowel diseases (IBD), the composition and diversity of the microbiota are always altered [3]. The imbalance between potentially “beneficial” and potentially “harmful” bacteria, also called dysbiosis, plays a role in the pathogenesis of chronic mucosal inflammatory lesions of IBD [4].

*F. prausnitzii* belongs to the phylum of Firmicutes and is the major bacterium of the* Clostridium leptum* group. The Meta-analysis of the Human Intestinal Tract project have shown that* F. prausnitzii* is one of the most abundant anaerobic bacteria in the human gut microbiota, with a proportion of around 5% of total bacteria in faeces [5].* F. prausnitzii* plays an important role in providing energy to the colonocytes and maintaining the intestinal health [6]. Furthermore, there is emerging laboratory evidence illustrating a strong anti-inflammatory effect of* F. prausnitzii* both in vitro and in vivo. And deficiency of* F. prausnitzii* might provoke and enhance inflammation [7]. Specially, a significant inverse correlation between disease activity and the count of* F. prausnitzii* was found in UC patients, even with quiescent disease [8]. The depletion of* F. prausnitzii* was observed in patients with untreated CD but not in the patients with chronic diarrhea, suggesting a relationship in the pathomechanisms of CD [9]. Finally, a diminished prevalence and abundance of* F. prausnitzii* are revealed in the fecal samples of patients with IBD. The* F. prausnitzii* level was much lower when the disease activity increased [10].

To further investigate the possible association between* F. prausnitzii* reduction and IBD, we conducted a meta-analysis and systematic review to estimate the relative risk of* F. prausnitzii* reduction in patients with and without IBD. Given the laboratory data previously cited, we hypothesized a relationship between* F. prausnitzii* reduction and IBD.

## 2. Materials and Methods

### 2.1. Search Strategy

This review was performed according to the standard guidelines for meta-analyses and systematic reviews of observational studies [11]. To find relevant articles for this review, we searched the following databases (from inception to November 2013): EMBASE, MEDLINE, Google Scholar, Pubmed, ACP Journal Club, the Cochrane Central Register of Controlled Trials, CMR, DARE, and HTA. The search strategy used free-text words to increase the sensitivity of the search. The following search terms were used: “inflammatory bowel disease,” “Crohn's disease,” “ulcerative colitis,” “IBD,” “UC,” “CD,” “*Faecalibacterium prausnitzii,*” “*F. prausnitzii,*” and “FP.” Boolean operators (AND, OR, NOT) were used to widen and narrow the search results. The titles and abstracts from the search results were examined for potential inclusion. Also, the references from selected articles were examined as further search tools.

### 2.2. Study Selection

For inclusion in the systematic review, a study had to meet the following criteria established by the study team: (1)* F. prausnitzii* counts measured by polymerase chain reaction-denaturing gradient gel electrophoresis (PCR-DGGE), terminal restriction fragment length polymorphism (T-RFLP), or fluorescence in situ hybridization (FISH), (2) studies of human, (3) inclusion of a control group, (4) IBD and control groups were similar in age and sex and from the same catchment area, and (5) data were reported that were sufficient to calculate* F. prausnitzii* reduction in both the IBD and control groups. Studies were excluded if they used data from a previously published study.

### 2.3. Data Extraction

To reduce reporting error and bias in data collection, 2 independent reviewers extracted data from selected studies using standardized data extraction forms. These forms, created by the study team, included the (a) title, (b) authors, (c) journal, (d) year of publication, (e) study design, (f) inclusion and exclusion criteria, (g) methods by which IBD was diagnosed, (h) methods by which* F. prausnitzii* reduction was diagnosed, (i) number of patients with ulcerative colitis (UC), (j) number of patients with Crohn's disease (CD), (k) number of patients in the control group, (l) reported previous use of antibiotics, probiotics, or prebiotics in the IBD and control groups, and (m) reported previous use of steroids, 5-aminosalicylates (5-ASAs), and tumor necrosis factor-alpha (TNF-*α*) antibody medications in the IBD group. Studies were excluded if participants had used steroids, 5-ASAs, TNF-*α* antibody antibiotics, probiotics, or prebiotics in the last month preceding fecal sampling as this could influence the intestinal microbiota. If needed, authors were contacted regarding specific questions relating to their study. The independent reviewers conferred after data extraction was complete, discrepancies were identified, and review of the relevant article led to consensus.

### 2.4. Statistical Analysis

The primary outcome of this analysis was the Standardization Mean Difference (SMD) of* F. prausnitzii* counts in IBD versus controls. Std. mean difference was used to describe the counts of the* F. prausnitzii* in IBD patients versus the controls. We calculated the SMD with a 95% confidence interval (CI) based on a random-effects model as described by Mantel-Haenszel. Meta-analysis was performed with the Review Manager 5.2. Analysis with a funnel plot used to assess publication bias. An *I* ^2^ statistic was used to measure the proportion of inconsistency in individual studies that could not be explained by chance. Any heterogeneity identified would prompt subgroup analysis in an attempt to explain these findings.

### 2.5. Assessment of Study Quality

Each study chosen for review was carefully assessed for study quality by the study team. Study quality was assessed using the following criteria: (1) study design, (2) method of IBD diagnosis, (3) method of patient enrollment (consecutive versus selected), (4) method of* F. prausnitzii* counts measurement, and (5) whether* F. prausnitzii* reduction was the primary or secondary outcome of the study.

## 3. Results

### 3.1. Search Results

**Figure 1:**
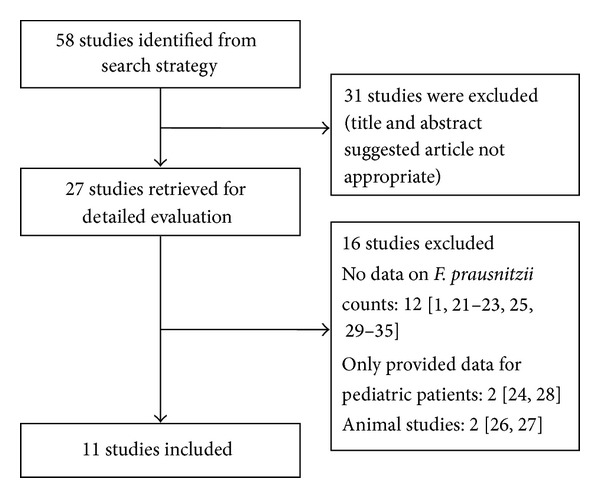
Flow diagram of studies identified in the systematic review.

Our initial search strategy yielded 58 potential articles for inclusion. After detailed analysis of selected articles, 27 articles were reviewed in detail. Subsequently, 16 articles did not meet inclusion criteria [1, 21–35]. The reasons for exclusion included: 12 studies did not provide data on* F. prausnitzii* counts [1, 21–23, 25, 29–35]. 2 studies only provided data for pediatric patients [24, 28]. 2 studies were animal studies [26, 27]. Therefore, 11 studies [8, 9, 12–20] with 1180 patients fulfilled the inclusion criteria for the review (Figure 1).

### 3.2. Study Characteristics

The characteristics of the included studies are summarized in Tables 1 and 2. The results of each study are in Table 3.* F. prausnitzii* counts were expressed as log10 values per gram feces. The largest and earliest study examining the relationship between* F. prausnitzii* reduction and IBD was conducted in Germany by Swidsinski et al. [13]. The authors investigated sections of paraffin-embedded punched fecal cylinders using fluorescence in situ hybridization (FISH).* F. prausnitzii* with high concentration was counted within a 10∗10 *μ*m area of the microscopic field representative of the region of interest.* F. prausnitzii* with uneven distribution or overall low concentrations was enumerated within larger areas of 100∗100 *μ*m.

8 of the included studies confirmed the differences in the presence or intensity of* F. prausnitzii* counts after denaturing gradient gel electrophoresis (DGGE) by real-time PCR (RT-PCR) [8, 12, 14–18, 20].* F. prausnitzii* cannot be cultured owing to its requirement for a complex anaerobic environment [19]. By RT-PCR, they were able to amplify, clone, and sequence the bacterial 16S ribosomal RNA genes and analyze the fecal samples individually to avoid the possible error [36].

Three of the included studies commented on the activity of IBD and* F. prausnitzii* counts. Wang et al. found sharply decreased* F. prausnitzii* in the feces of active CD and UC patients [16]. Sokol et al. and Andoh et al. reported lower counts of* F. prausnitzii* in active CD patients compared to CD patients in remission [18, 19].

1 of the included studies examined the* F. prausnitzii* counts before and after treatment by an element diet [17]. It suggests recovery following elemental diet is attributed to lower levels of gut flora.

1 of the included studies reported the relationship between the maintenance of clinical remission and the recovery of the* F. prausnitzii* population after relapse Varela et al. found low counts of* F. prausnitzii* were associated with less than 12 months of remission and more than 1 relapse/year [12].

### 3.3. Meta-Analysis of SMD

**Figure 2:**
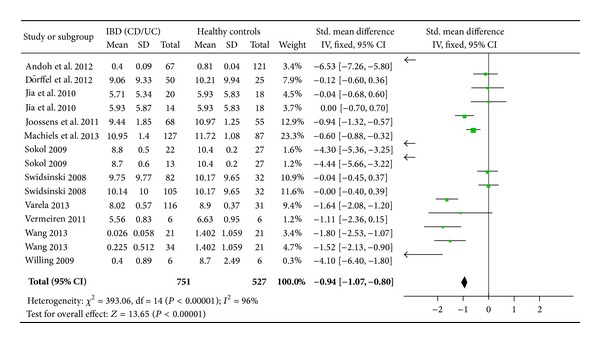
Forest plot of rate of* F. prausnitzii* reduction in patients with IBD versus controls.

**Figure 3:**
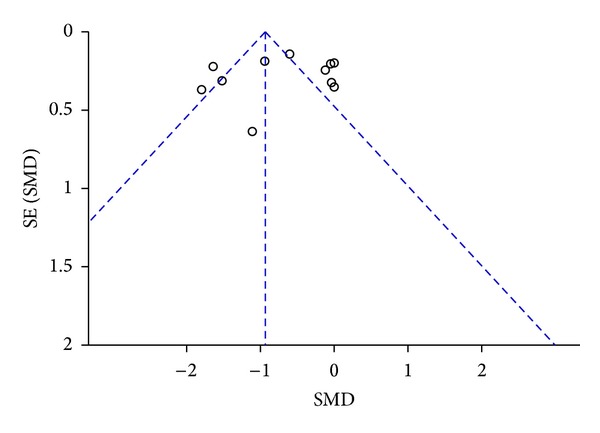
Funnel plot analysis.

Overall, the bacterial count of* F. prausnitzii* in IBD patients was significantly lower (6.7888 ± 1.8875) log10 CFU/g feces than healthy controls (7.5791 ± 1.5812) log10 CFU/g feces; *P* < 0.0001. The SMD of* F. prausnitzii* in IBD patients was −0.94 (95% confidence interval [CI]: −1.07–−0.80) (Figure 2). Subgroup analyses revealed a trend toward a greater effect for CD (SMD: −1.13, 95% CI: −1.32–−0.94) when compared to UC (SMD: −0.78, 95% CI: −0.97–−0.60). There was significant heterogeneity in the included studies (*I* ^2^ = 96%). Furthermore, analysis of the funnel plots for publication bias suggested a possible bias against small studies demonstrating high SMD (Figure 3).

## 4. Discussion

Our systematic review and meta-analysis of the literature has identified recent studies examining the relationship between* F. prausnitzii* reduction and IBD. The majority of recent studies find a higher rate of* F. prausnitzii* reduction in IBD patients as compared to controls. All of the 11 included studies found significantly lower* F. prausnitzii* counts in IBD patients versus controls. Our meta-analysis suggests a possible link with the reduction of* F. prausnitzii *andmisbalance of the intestinal microbiota and IBD patients, especially CD patients with ileal involvement. The levels of* F. prausnitzii* were extremely low in two studies [19, 28]. Wang et al. took biopsies samples from active CD patients and found extremely lower* F. prausnitzii* counts compared to stools [28]. Andoh et al. demonstrated a difference in gut microbiota of the Japanese population, suggesting that environmental factors such as sanitation, diet, hygiene, and ethnicity were important for shaping the gut microbiota [19]. However, significant heterogeneity and the possibility of publication bias limit our certainty in this association. Furthermore, Hansen et al. challenged the current model of a protective role for* F. prausnitzii *in CD [24]. They reported an increasein mucosal* Faecalibacterium* in pediatric CD patients compared with controls, which suggested a more dynamic role for this organism than previously described in adult IBD. It is possible that the microbial signature of pediatric IBD is distinct from adult disease. Furthermore, the early host and microbiota response to IBD may induce proliferation of* F. prausnitzii* to reverse the inflammatory change, which still remains to be explained.

Mechanistic theories of microbial etiopathogenesis between the possible protective benefit of* F. prausnitzii* against IBD have been proposed. Duncan et al. demonstrated that the major end products of glucose fermentation by* F. prausnitzii* strains are substantial quantities of butyrate [37]. Butyrate plays a major role in gut physiology, protection against pathogen invasion, and modulation of immune system [38]. Butyrate is the primary energy source for intestinal epithelial cells, which are fundamental elements for the maintenance of barrier integrity [26]. Therefore, butyrate may contribute to the anti-inflammatory effect. Additionally, butyrate may inhibit inflammatory response through inhibition of histone deacetylase activity, resulting in suppression of NF-*κ*B activity and hyperacetylation of histones [12]. Furthermore, Himmel et al. found that* F. prausnitzii* could induce relatively low amounts of IL-12 and large amounts of IL-10 and Tregs in epithelial and PBMC models to restrain the progression of inflammation [39]. While Sokol et al. reported that* F. prausnitzii *led to significantly lower IL-12 and IFN-*γ* production levels and higher secretion of IL-10 in vitro peripheral blood mononuclear cells [26].

The data on the incidence of* F. prausnitzii* reduction and IBD found in the literature has several limitations. Most of the studies did not comment on the participants' previous history of treatment such as antibiotics, probiotics, or prebiotics which may influence the intestinal microbiota. It is therefore possible that study participants had been treated for dysbiosis prior to entering the study, thereby producing a false* F. prausnitzii* reduction. Additionally, we did not evaluate confounding factors including diet and smoking in our study, even though these factors might reduce* F. prausnitzii* levels and faecal butyrate values [40]. Furthermore, some of the included studies did not clearly identify the criteria for the IBD diagnosis. Few commented on personal review of the endoscopic findings or histology. Also we did not relate the differences in microbiota to geography and ethnicity. Lastly, most studies were performed at a single medical center.

Future studies should address these limitations. After confirming the diagnosis of IBD through the endoscopic and histological findings, PCR-DGGE, T-RFLP, or FISH for* F. prausnitzii* counts would be initiated. In patients found to have* F. prausnitzii* reduction, probiotics or prebiotics may be used to restore the “ecological balance” of intestinal microbiota. Dörffel et al. reported rifaximin was associated with an increased level of* F. prausnitzii* [9]. Other specific treatments such as infliximab and a high-dose cortisol therapy were shown to reverse the depletion of* F. prausnitzii* [13]. The mechanism for the inverse association between* F. prausnitzii* reduction and the initiation and perpetuation of inflammatory bowel disease has yet to be defined. Healthy controls who are age- and sex-matched to the IBD group would be selected from the same area as the IBD group and tested for* F. prausnitzii* by the same method. In both groups, a thorough history examining previous treatment such as antibiotics, probiotics, or prebiotics, steroids, 5-aminosalicylates (5-ASAs), and tumor necrosis factor alpha (TNF-*α*) antibody would be obtained. Prideaux et al. reported that regardless of ethnicity or geography, Crohn's disease resulted in reduced bacterial diversity. However, in ulcerative colitis, diversity was reduced in Chinese subjects only. It suggested that ethnicity might also play an important role in the pathogenesis of IBD [41].

## 5. Conclusions

In summary, our meta-analysis and systematic review suggest a possible protective benefit of* F. prausnitzii* against the development of IBD. However, significant variation among the studies and the possibility of publication bias limit the certainty of this association. Therefore, further treatment such as probiotics or prebiotics to increase the levels of* F. prausnitzii* in IBD are lead to attempts. If* F. prausnitzii* is found to indeed protect against IBD, we can approach the treatment such as supplementing the microorganisms that produce butyric acid.

## Figures and Tables

**Table 1:** Characteristics of the included studies.

Author	Year	Location	Single versus multicenter	*n*, total	*n*, IBD (CD/UC)	*n*, control	Control composition	Mean age, IBD (CD/UC)	Mean age, control
Machiels et al. [8]	2013	Belgium	Single	214	0/127	87	Healthy controls	43	41.5
Varela et al. [12]	2013	Spain	Single	176	0/116	31	Healthy controls	40	32
Swidsinski et al. [13]	2008	Germany	Single	422	82/105	32	Healthy controls	35/41	40
Dörffel et al. [9]	2012	Germany	Single	171	50/0	25	Healthy controls	39	48
Vermeiren et al. [14]	2012	Belgium	Single	12	0/6	6	Healthy controls	Not reported	Not reported
Joossens et al. [15]	2011	Belgium	Single	207	68/0	55	Healthy controls	Not reported	Not reported
Wang et al. [16]	2013	China	Single	76	21/34	21	Healthy controls	Not reported	Not reported
Jia et al. [17]	2010	UK	Single	73	20/14	18	Healthy controls	Not reported	Not reported
Sokol et al. [18]	2009	France	Single	133	22/13	27	Healthy controls	37/40	36
Andoh et al. [19]	2012	Japan	Multicenter	188	67/0	121	Healthy controls	30	32
Willing et al. [20]	2009	Sweden	Single	20	6/0	6	Healthy controls	Not reported	Not reported

IBD: inflammatory bowel disease; CD: Crohn's disease; UC: ulcerative colitis.

**Table 2:** Quality assessment of the included studies.

Author	*F. prausnitzii* counts	IBD diagnosis	Study type	Patient enrollment	Outcome	Samples
Machiels et al. [8]	RT-PCR	Not reported	Retrospective	Not reported	Primary	Stools
Varela et al. [12]	RT-PCR	Colonoscopy and histology	Retrospective	Not reported	Primary	Stools
Swidsinski et al. [13]	FISH	Colonoscopy	Retrospective	Not reported	Primary	Stools
Dörffel et al. [9]	FISH	Colonoscopy	Retrospective	Not reported	Primary	Stools
Vermeiren et al. [14]	RT-PCR	Not reported	Retrospective	Not reported	Primary	Stools
Joossens et al. [15]	RT-PCR	Not reported	Retrospective	Not reported	Primary	Stools
Wang et al. [16]	RT-PCR	Not reported	Retrospective	Not reported	Primary	Biopsies
Jia et al. [17]	RT-PCR	Not reported	Retrospective	Not reported	Primary	Stools
Sokol et al. [18]	RT-PCR	Not reported	Retrospective	Not reported	Primary	Stools
Andoh et al. [19]	T-RFLP	Not reported	Retrospective	Not reported	Primary	Stools
Willing et al. [20]	RT-PCR	Colonoscopy	Retrospective	Not reported	Primary	Biopsies

**Table 3:** Study results.

Author	log⁡10 copies/g IBD patients (CD/UC)	log⁡10 copies/g healthy controls	*P*
Machiels et al. [8]	0/(10.95 ± 1.41)	11.72 ± 1.08	<0.0001
Varela et al. [12]	0/(8.02 ± 0.57)	8.90 ± 0.37	<0.0001
Swidsinski et al. [13]	(9.75 ± 9.77)/(10.14 ± 10.02)	10.17 ± 9.65	<0.0001
Dörffel et al. [9]	(9.06 ± 9.33)/0	10.21 ± 9.94	<0.001
Vermeiren et al. [14]	0/(5.56 ± 0.83)	6.63 ± 0.95	0.07
Joossens et al. [15]	(9.44 ± 1.85)/0	10.97 ± 1.25	<0.0001
Wang et al. [16]	(0.03 ± 0.06)/(0.23 ± 0.51)	1.40 ± 1.06	<0.0001
Jia et al. [17]	(5.71 ± 5.34)/(5.93 ± 5.87)	5.93 ± 5.83	<0.05
Sokol et al. [18]	(8.81 ± 0.52)/(8.70 ± 0.63)	10.4 ± 0.2	0.0004
Andoh et al [19]	(0.40 ± 0.09)/0	0.81 ± 0.04	<0.0001
Willing et al. [20]	(0.40 ± 0.89)/0	8.72 ± 2.49	<0.001
